# Miscellaneous Notices

**Published:** 1850-04-01

**Authors:** 


### Miscellaneous Notices.

*Evening Thoughts.* By A Physician. 1850.

We are not acquainted with any profession to which the great problem of human existence is presented in so many variously stamped features as the medical. It waits on man at his birth, and ever watchful in the whole course of his after existence, whenever the mortal and immortal parts have received some violent shock, it carefully guides him to the haven of health, or waits in solemn stillness, until the last scene closes all—returning his body to its kindred dust—and the spirit to his Maker—to the bosom of his God. Engaged in those solemn duties of humanity, it cannot otherwise happen than that the mind should be deeply impressed with the consideration of those great and solemn questions which affect the individual man—life, death, eternity, immortality, and all other kindred topics relating specially to the higher endowments of the human being.

The volume before us embraces a succession of detached thoughts on different subjects relating to man:—*Life—Religion—The Soul—Spiritual Analogies—The Will—The Heart—Philosophy* and *Christianity—The Invisible—Evil—Light—Substance—Spiritual Sense Organ—The Open Secret—Systematizing Divines, &c. &c. &c.* They are written in a plain style, always clear and explicit, but not always devoid of Germanisms, nor happily adjusted in the sequences of the sentences, or even of the clauses—faults comparatively of a trivial character, as the perspicuity is never sacrificed, and obviously arising from the condensed and almost epigrammatic manner in which they have been of necessity developed.

They are evidently the production of a mind at once reflective and capable of exciting reflection. They bespeak no ordinary width of reading and range of information, and they are obviously the result of much careful thought and studious reflection, displayed equally in the new relations in which he discusses the different topics as they are successively marshalled in the field of observation, as well as in the original suggestions of his own mind. His knowledge of the seers of ancient philosophy seems to be most ample, and his acquaintance with those of more recent date not less satisfactory. The sole empire of sensation he unreservedly disclaims, and even is unwilling to admit the claims of the phrenologists, rather on the assumption that their doctrine has a distinct tendency to the theory of Materialism, than from any substantial direct argument he has to offer against their tenets. Though the reflections are decidedly of English growth, they are tinctured throughout with no inconsiderable bias towards the German school of philosophy, whose ultra-transcendentalism, be it attentively noted, he rather eschews, mantling, nevertheless, the general tenour of his observations by a sound, and withal refreshing estimate of the importance of religion.

The thoughts at eventide have this one further excellence: they excite thought, and, at the same time, are suggestive of a mode by which we may in our own persons practically test the truth of some of the great problems they involve.

In concluding the summary notice of a work, which (nor is it the least of its merits) is decidedly of a most awakening spirit, and which has been conceived in a liberality of feeling honourable alike to the author's head and heart, we are led to express a hope that he may be induced to extend his propositions, for they are rather to be considered as corollaries, than an extended and amply elucidated disquisition, on the respective subject-matters which they profess to handle. Much of the topics he has touched on, are of a nature too recondite to be apprehended by the many; and the most enlightened will concede, that there are some of them which as yet we see but through a glass darkly; nevertheless, as the shadow is, however unsubstantial in reality, the indication of an *essence*, what is seen *darkly*, is equally the type of an essence, though unknown and invisible. That the finite shall ever comprehend the infinite—the created the Creator, we have not the arrogance to presume, though we judge much of the so-called philosophy of the day is an attempt to develop and disclose the great system of the creation. Time, however, will gradually dispel much of that flimsy sophistry which has hitherto held the human mind in thraldom, and given it the false conceit of solving the philosophy of human nature in the inflated terms—"*Par Ingenium Naturæ.*" But as the late accomplished philosopher, Sir James Smith, quietly but aptly withal observed, "a blade of grass was sufficient to baffle the world-making fancies of a Buffon." Thus we would further observe, that in an age so replete with every kind of knowledge, it would seem strange if the philosophy of the mental and physical relations were not better understood; for on the arid physical theory, while everything else created has a probable solution, "man, the paragon of animals," is yet unsolved.

"Then he of all that Nature has brought forth,
Stands self-impeached, the creature of least worth:
And useless when he lives, and when he dies,
Brings into doubt the wisdom of the skies."

*The Anti-Materialist denying the Reality of Matter, and vindicating the Universality of Spirit.* By John Dudley, Clerk, Author of "Naology." London: Bell. 1849.

Superstition and Atheism, Credulity and Scepticism, are the antagonistic principles of the intellectual and moral world. There are Platonists among us who have an unbounded faith in Spiritualism, and Pyrrhonists who dispute the existence of the earth on which they tread. This diversity—or rather conflict of opinion, is as necessary and essential to the interest of truth as the agitation of the sea is to the purity of its waters. Absolute unanimity of opinion would be fatal to our intellectual and social progress;—truth and error, therefore, are alternating links in the same chain. Hence, out of the rank soil of Materialism sprung the fascinating philosophy of Idealism. The sceptical doctrines propounded by Hobbes gave rise to the rationalism of Locke; and the antagonism he met with suggested the idealistic principles which Bishop Berkeley and Collier simultaneously hazarded. The war between Materialists and non-Materialists soon raged with the same intensity as in after years distinguished the geological fray between the Vulcanists and the Neptunians—between the Huttonians and the Wernerists. Much ink was shed, and the shafts of wit and sarcasm, like poisoned arrows, were discharged in all directions. But after all, was it not a contest of words only—mere paradoxical quibbling, arising out of an attempt to discover something infinitely more recondite than the philosopher's stone? "Where are we to discover the *substratum* of matter?" asked the Idealist—"take away its properties and *where* is it?" True! We are writing at a goodly-sized round table—take away its extension, its solidity, its form, and the table would vanish from before us as certainly as if it dropped through the stage-trap of a Christmas pantomime. But the objective reality of these collective properties—its solidity, roundness, colour, &c., constituting it a visible and tangible table, cannot, we apprehend, be denied. Hence, even Bishop Berkeley admitted the existence of a phenomenal world; and we can hardly conceive any one denying what Reid designated a primary and fundamental law of human belief.

The author before us, Mr. Dudley, avows himself, however, not only an anti-materialist, but, in the course of his work, he out-Berkeleys Berkeley himself, and so wide and universal is his spiritualism that he wanders far and wide beyond the boundaries of the present world. We cannot conceive a finite being—and we presume Mr. Dudley is one—speculating on such subjects as the following:—"How the Deity may possibly have been employed before creation was begun." (Anti-Materialist, page 20.) "How the good and evil angels must of necessity have waged none other than a spiritual warfare." (Ibid, page 85.) "How heaven and hell are not material localities, but spiritual conditions." (Ibid, page 86.) These, and conjectures as dreamy, are here conjured up, and carry the imagination far beyond the sphere of legitimate philosophy. It is an acknowledged and trite axiom that a finite being cannot understand that which is infinite; "How wonderful," exclaimed the dormouse, emerging from underneath a grotto, "must have been the dormouse that built this temple." And man, with his limited faculties, speculating on the nature and will of the Deity, is equally at fault. The author not only disbelieves in the existence of matter, but he affirms that "all the errors, absurdities, and impurities which have in different ages of the world marred the interests, disturbed the peace, and corrupted the piety of mankind, have been occasioned, more or less directly, by the opinion of the reality of matter." (Page 13.) Here, then, we have a clue by which we may explain the imposition of Johanna Southcott, and the causes of the late French Revolution. Men believed in the existence of matter, and this appearance of matter led their wits astray: in avoiding Scylla, how easy it is for the imagination to wreck itself on Charybdis. But why should the author peril his faith on such a sweeping dogma as this? The most learned philosophers and divines agree in believing that there is a material and an immaterial world; and that man is a being endowed with body and soul. But our antimaterialist will not have this doctrine upon any terms, and even goes so far as to declare that when "the Saviour became flesh he did not become a material body." Assuredly this is an heterodox notion, which, if once admitted, would undermine the foundation of Christianity.

We by no means wish to deal harshly with the author, for his book displays much learning and argumentative ingenuity, but we must suggest to him that such discussions are very unprofitable and unwise, and carry us back to the scholastic reveries and darkness of the middle ages.

*A Practical Treatise on Morbus Coxarius, or Hip-joint Disease.* By WILLIAM C. HUGMAN, M.R.C.S., Surgeon to the Verral Institution. S. Highley.

THE purport of the work is to bring before the profession a new method of treatment by mechanical means, which, judging from the very satisfactory results obtained by the author's practice, must fairly be considered well worthy the attention of surgeons generally. It cannot be doubted that, under the ordinary methods of treatment hitherto employed, these cases seldom terminate without a considerable amount of deformity, owing in a great measure to the malposition assumed by the patient in bed during the tedious course of the disease. The author professes to have conquered this serious evil by means of the couch he employs, which also relieves the pain and inflammation of the joint by the removal of all pressure from the affected part. His views of the pathology of the disease are plausible, and concisely given. The internal remedy he considers of most value in the majority of cases is the cod-liver oil. The monograph throughout is written in a professional spirit, and its character justifies us in recommending it.

